# Prevalence of mastitis in dairy goat farms in Eastern Algeria

**DOI:** 10.14202/vetworld.2019.1563-1572

**Published:** 2019-10-15

**Authors:** Zahra Gabli, Zouhir Djerrou, Abd Elhafid Gabli, Mounira Bensalem

**Affiliations:** 1 Department of Nature and Life Sciences, Faculty of Sciences, University of August 20^th^ 1955, Skikda, Algeria; 2 Laboratory of Pharmacology and Toxicology, University of Mentouri Constantine 1, Algeria; 3 Department of Hygiene and Animal Health, Institute of Veterinary Sciences, University of Mentouri Constantine 1, Algeria

**Keywords:** Algeria, bacteriological analysis, California mastitis test, dairy goats, mastitis

## Abstract

**Aim:**

This study aimed to investigate mastitis in dairy goat farms through the California mastitis test (CMT) and bacteriological examinations

**Materials and Methods:**

A total of 845 goats belonging to 18 farms from four regions (Tébessa, Guelma, Souk Ahras, and Skikda) were examined.

**Results:**

Clinical examination of the mammary glands showed that 30/845 (3.55%) goats had clinical mastitis and 32 goats had half-teat inflammation. CMT subclinical mastitis (SCM) was detected in 815 goats that were presumed to be healthy. CMT showed 46 (5.64%) CMT-positive goats as well as 47 (2.88%) positive half-udders with a score of ≥2. A total of 79 bacteria were isolated and identified from the 79 bacterial positive samples. Bacteriological analyses showed that Gram-positive staphylococci were largely responsible for clinical and SCM. Coagulase-negative staphylococci, with an isolation frequency of 56.96%, were the most prevalent bacteria from all isolated organisms. The second most prevalent organism was *Staphylococcus aureus* at 40.50% and streptococci (2.53%) had the smallest percentage of isolation.

**Conclusion:**

It is suggested that due to the prevalence of mastitis in this species, farmers should be aware of the problem to plan preventive and control measures to reduce dairy goat losses due to this disease.

## Introduction

Mastitis is a general term that refers to the inflammation of mammary tissues, without regarding the cause of the inflammation [1]. It has a number of adverse health effects that affect animal welfare, agricultural economy, and food security [2,3].

Mastitis is traditionally classified into clinical and subclinical divisions. Subclinical mastitis (SCM) is the most common form of mastitis, with prevalence in about 15-40% of intramammary infected dairy goats. It is a form of mastitis without the typical clinical signs of an infected mammary gland, but the pathogens still exist in the milk and colonize the mammary gland [4].

Milk produced by goats with mastitis presents a serious risk in terms of public health as it can be linked to milk-borne diseases for humans. In addition to causing hygiene and health problems, inflammation of the mammary glands can also cause economic losses due to reduced milk production, poor quality of milk, early culling of animals, and increased treatment costs [3,5,6].

Mastitis is considered one of the most important diseases in domestic animals caused by multiple etiological agents. However, *Staphylococcus aureus* is considered to be the most common causative agent of goat mastitis, followed by a minor occurrence of mastitis by *Pasteurella haemolytica*, *Escherichia coli*, *Clostridium perfringens*, *Streptococcus*, *Pseudomonas*, and *Nocardia* genera [7].

The diagnosis of mastitis is a tedious task and can be performed using the California mastitis test (CMT) and a somatic cell count (SCC). In addition, bacteriological isolation and polymerase chain reaction identification can also be performed to confirm diagnosis [8].

Bourabah *et al*. [9] investigated the periodic prevalence and etiology of SCM in goats in the Tiaret region (Western Algeria). However, to the best of our knowledge, there have been no studies yet on goat mastitis in the four regions (Tébessa, Souk-Ahras, Guelma, and Skikda) of Eastern Algeria. This study aimed to determine the prevalence and the causative bacteria of mastitis in dairy goat farms in the four Eastern Algeria regions.

## Materials and Methods

### Ethical approval

The design of the present study was approved by the ethical committee of the Institute of Veterinary Sciences, University Mentouri Constantine 1, Algeria.

### Study areas and animals

This study was conducted on 845 Mekatia local dairy goats (142 primiparous and 703 multiparous) belonging to 18 extensive goat farms located in four regions of Northeastern Algeria. Two regions (Tébessa and Souk Ahras) are located in the steppe highlands. These regions are characterized by a semi-arid climate and an altitude of more than 1200 m. Rainfall varies from 200 to 350 mm/year. As a result, there is a large thermal amplitude, winter frosts range from −2°C to 4°C, and strong summer heat from 33°C to 38°C. The other two regions (Guelma and Skikda) are characterized by a rainy season from October to April and a dry season from May to September. The minimum temperature is 7-15°C in the winter and the maximum temperature is 32-42°C in the summer. The altitude varies between 800 m and 1200 m. These geoclimatic features are favorable for the development of goat farming. This study took place from January 2015 to March 2018 and involved goats aged 2-6 years, between the 2^nd^ and 6^th^ months of their lactation. The number of goats examined varied from 30 to 60 lactating goats per farm. The animals were free from brucellosis and were not subject to any antibiotic treatment at sampling.

### Clinical mastitis

#### Clinical examination of the udder

A total of 845 goats were clinically examined. After a thorough visual examination, we palpated each one of the udder halves. The aim was to identify any signs of mastitis (inflammation that results in a red, hot, swollen, hard, and/or painful udder). Changes in milk color and consistency and general symptoms, such as fever, depression, and/or loss of appetite, were also assessed [10-12].

### SCM

#### Screening for SCM by CMT

A total of 815 of 845 presumed healthy goats were collected before the morning milking and carried out from the 3^rd^ week of lactation to avoid interference with the colostral period [13]. The milk samples from the first jet of each quarter were put in a clean cup, and we poured the overflow to retain only about 2 ml milk per quarter. We then added an approximately equal amount of surfactant reagents based on Teepol (sodium alkyl aryl sulfate), which induced reagents for cell membrane lysis and nuclear DNA precipitation. From this, a gel was then formed [14,15]. CMT was then performed and the results were assessed according to the manufacturer’s recommendations (Table-1) [11]. A milk sample was microbiologically assayed when CMT value was ≥2.

**Table 1:** CMT interpretation scale (David and De Cremoux, 2000).

Appearance of the gel	Interpretation	Note
No precipitation	Negative	0
Cloudy precipitation, which disappears	Trace	1
Light persistent gel with lumpy filament	Doubtful	2
Immediate thickening, “egg white” type gel, detaching from the bottom in filament during rotation of the tray	Slightly positive	3
Curved gel, sliding in mass on the bottom of the plate during its rotations	Very positive	4

CMT=California mastitis test

### Sampling and bacteriological examination of mastitis milk

All inflamed halves and halves with positive CMT were sampled just before the morning milking. A volume of 10 ml of milk from each quarter was collected in a sterile glass tube after teat disinfection with alcohol at 70°, washing, and removal of the first jet. The samples were labeled and immediately sent to the laboratory of the Institute of Veterinary Sciences, El Khroub, in a refrigerated cooler and placed in the refrigerator at +4°C. All milk samples collected from mastitis (clinical and subclinical) were subjected to bacteriological analysis in the laboratory using standard testing procedures as described in other studies [16]. Cultural bacteriology remains the reference method in the etiological diagnosis of mastitis in goats. It is a diagnosis of “certainty” highlighting the presence of a bacterium in milk. The bacteriological isolation of milk was carried out by depositing 10 μl of each sample on sheep blood agar medium (5%). After incubation for 24-48 h at 37°C, the dishes were examined for the presence and appearance of bacterial colonies. Bacteria identification was performed through conventional methods (colony appearance, Gram stain, catalase test, and bound coagulase test). The biochemical characters were studied through API 20 tests (API bioMérieux, France), allowing the characterization of bacterial species within the same genus [17].

## Results

### Clinical mastitis

#### Clinical examination of the udder

**Figure-1:**
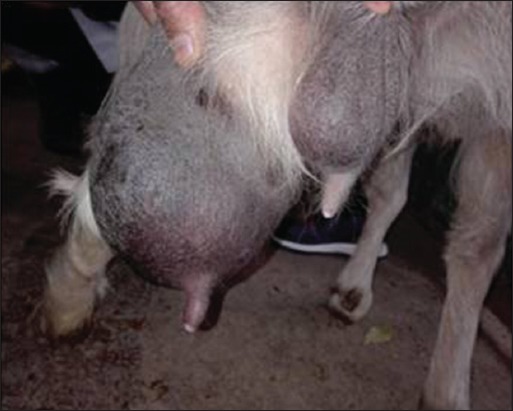
Asymmetry and inflammation of the udder pair in the goat.

**Figure-2:**
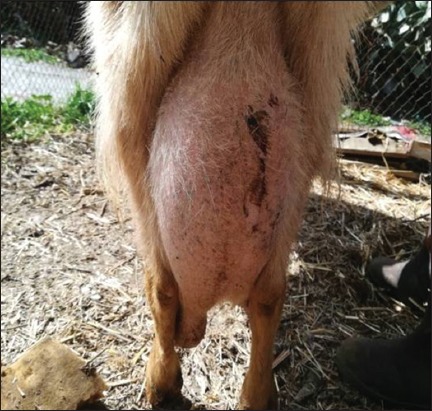
Infected left half-udder of the goat.

**Figure-3:**
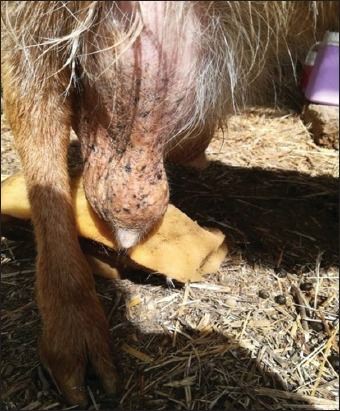
Infected right half-udder of the goat.

Clinical examination of the mammary glands of 845 dairy goats revealed 30 (3.55%) goats with clinical mastitis, of which 6/30 (20%) were primiparous and 24/30 (80%) were multiparous. Of the 30 goats, 28 (93.34%) goats had only half of their udder affected, while both teats were affected in the remaining 2 goats (6.66%). We have selected a few cases of clinical mastitis (Figures-1-3). This survey shows that out of the surveyed farms, infection was present in 16/18 farms (88.88%), and 2/18 farms (11.11%, H and J) were free of intramammary infection (Table-2).

**Table 2:** Results of bacteriological analyses of clinical mastitis milk in the four regions.

Regions	Number of examined goats	Breeders	Number of goats per breeder	Number of primiparous and multiparous examined		Burning neighborhoods		Bacteriological analyses			Number of isolated germs by region	Number of infected goats by region		
	
						One neighborhood	Two neighborhoods	Isolated germs			
	
								*Stap.aureus*	*Strep.* spp.			P	M	P+M (%)

									*Strep.uberis*	*Strep.agalactiae*				
Tébessa	236	A	37	P	8	-	0				11/32 (34.37%)	2	8	10/236 (4.23)
				M	29	-	1 (1×2)	2						
		B	60	P	15	1	0	1						
				M	45	2	0	2						
		C	46	P	12	-	0							
				M	34	1	0	1						
		D	51	P	19	1	0	1						
				M	32	1	0		1					
		E	42	P	10	-	0							
				M	32	3	0	3						
Souk Ahras	280	F	56	P	15	1	0	1			6/32 (18.75%)	1	4	5/280 (1.78)
				M	41	1	0	1						
		G	49	P	13	-	0							
				M	36	1	0	1						
		H	48	P	14	-	0							
				M	34	-	0							
		I	30	P	5	-	0							
				M	25	-	1 (1×2)	2						
		J	59	P	16	-	0							
				M	43	-	0							
		K	38	P	9	-	0							
				M	29	1	0	1						
Guelma	189	L	54	P	13	-	0				7/32 (21.87%)	1	6	7/189 (3.70)
				M	41	2	0	2						
		M	40	P	8	1	0	1						
				M	32	1	0	1						
		N	38	P	7	-	0							
				M	31	1	0	1						
		O	57	P	14	-	0							
				M	43	2	0	2						
Skikda	140	P	58	P	16	-	0				8/32 (25%)	2	6	8/140 (5.71)
				M	42	2	0	2						
		Q	49	P	14	1	0	1						
				M	35	2	0	1		1				
		R	33	P	5	1	0	1						
				M	28	2	0	2						
Total	845	18	845	P	213	6	0	30/32 (93.76%)	1/32 (3.12%)	1/32 (3.12%)	32	6/213 (2.81%)	24/632 (3.79%)	30/845 (3.55%)
				M	632	22	4 (2×2)							
				P+M	845	32								

P=Primiparous, M=Multiparous, *Stap.*=*Staphylococcus, Strep.*=*Streptococcus*

#### Bacteriological examinations of clinical mastitis milk

In this investigation, we analyzed 32 milk samples collected from 30 goats with clinical mastitis. The cultures were monobacterial with only one dominant species, whether it was an infected quarter or two. Thirty-two bacteria were isolated from the samples and divided into three different bacterial species.

*S. aureus* was the predominant species as it was present in 30/32 (93.75%) of the samples. Streptococci were isolated with a frequency of 2/32 (6.25%), of which 1/32 (3.12%) was *Streptococcus uberis* and 1/32 (3.12%) was *Streptococcus agalactiae*.

The distribution of isolated germs varies from one region to another. The highest number was recorded in the Tébessa region, where 11/32 (34.37%) species were isolated. The next highest were Skikda with 8/32 (25%), Guelma with 7/32 (21.87%), and Souk Ahras with 6/32 (18.75%) (Table-2). On the other hand, the level of infection varied according to the region. We recorded frequencies in descending order: Skikda with 5.71% (8/140), Tebessa with 4.23% (10/236), Guelma with 3.70% (7/189), and Souk Ahras with 1.78% (5/280). Finally, the total frequency of the four regions was 3.55% (30/845), of which 6/213 (2.81%) were primiparous and 24/632 (3.79%) were multiparous (Table-2).

### SCM

#### CMT test

CMT was performed on 815/845 healthy presumed goats belonging to 18 farms. The results of individual CMTs revealed 46 (5.64%) goats with SCM, of which 13/207 (6.28%) were primiparous and 33/608 (5.42%) were multiparous. In addition, 47 half-udders had CMT score of ≥2 and 45/47 half-udders were from one neighborhood where one udder or both quarters were affected. The level of the positivity of the test was 6.19% (14/226) for Tébessa, 4.36% (12/275) for Souk Ahras, 6.04% (11/182) for Guelma, and 7.57% (9/132) for Skikda (Table-3).

**Table 3:** Distribution of CMT results from presumed healthy goats and bacteriological analyses of CMT-positive milk in the four regions.

Regions	Number of examined goats	Breeders	Number of healthy goats by breeding	Number of goats taken for CMT		Positive neighborhoods at CMT (score ≥2)		Bacteriological analyses								Number of isolated germs by region	Number of infected goats per region		

								Isolated germs								
		
						One neighborhood	Two neighborhoods	Coagulase-negative staphylococci									P	M	P+M (%)

								*Stap. caprae*	*Stap. xylosus*	*Stap. simulans*	*Stap. epidermidis*	*Stap. cohnii*	*Stap. lentus*	*Stap. hominis*	*Stap. aureus*				
Tébessa	226	A	36	P	8	0										14/47 (29.78%)	3	11	14/226 (6.19)
				M	28	2		1		1									
		B	57	P	14	1		1											
				M	43	2		1							1				
		C	45	P	12	1				1									
				M	33	2				1	1								
		D	49	P	18	1					1								
				M	31	2		1	1										
		E	39	P	10	0													
				M	29	3		1		1			1						
Souk Ahras	275	F	54	P	14	1			1							13/47 (27.65%)	3	9	12/275 (4.36)
				M	40	1				1									
		G	48	P	13	0													
				M	35	1		1											
		H	48	P	14	0													
				M	34	1					1								
		I	29	P	5	1		1											
				M	24	1						1							
		J	59	P	16	0													
				M	43	2		1	1										
		K	37	P	9	1		1											
				M	28	2	1 (1×2)	2	1				1						
Guelma	182	L	52	P	13	0										11/47 (23.40%)	4	7	11/182 (6.04)
				M	39	1									1				
		M	38	P	7	2		1			1								
				M	31	2		1	1										
		N	37	P	7	1		1											
				M	30	2			1			1							
		O	55	P	14	1		1											
				M	41	2				1				1					
Skikda	132	P	56	P	16	1					1					9/47 (19.14%)	3	6	9/132 (7.57)
				M	40	2		2											
		Q	46	P	13	1				1									
				M	33	1			1										
		R	30	P	4	1			1										
				M	26	3		1				2							
Total	815	18	815	P	207	13	0	18/47 (38.2%)	8/47 (17.02%)	7/47 (14.89%)	5/47 (10.63%)	4/47 (8.51%)	2/47 (4.25%) 45	1/47 (2.12%)	2/47 (4.25%)	47	13/207 (6.28%)	33/608 (5.42%)	46/815 (5.64)
				M	608	32	2 (1×2)												
				P+M	815	47													
							45/47(95.75%)												

P=Primiparous, M=Multiparous, *Stap.*=*Staphylococcus, Strep.*=*Streptococcus*, CMT=California mastitis test

#### Bacteriological examinations of milk positive for CMT

A total of 47 samples of milk positive for CMT showed 47 bacteria divided into eight different bacterial species with a predominance of *Staphylococcus caprae* at 18/47 (38.29%), followed by *Staphylococcus xylosus* at 8/47 (17.02%), *Staphylococcus simulans* at 7/47 (14.89%), *Staphylococcus epidermidis* at 5/47 (10.63%, *Staphylococcus cohnii* at 4/47 (8.51%), *Staphylococcus lentus* at 2/47 (4.25%), *S. aureus* at 2/47 (4.25%), and *Staphylococcus hominis* at 1/47 (2.12%). Our investigation reveals the importance of coagulase-negative staphylococci (CNS) as the most dominant germs at 45/47 (95.75%) versus *S. aureus* which was isolated at a low-frequency rate of 2/47 (4.25%). The frequency of isolated organisms varies from one region to another: Tébessa at 29.78% (14/47), Souk Ahras at 27.65% (13/47), Guelma at 23.40% (11/47), and Skikda at 19.14% (9/47). This bacteriological study shows that SCM exists in all farms. The distribution of different species of isolated bacteria is shown in Table-3.

### Distribution and frequency of bacterial species isolated from mastitis

Only the bacteriological diagnosis was used to characterize the infections of the half-udders in this study. The results of the bacteriological analyses revealed 79 bacterial species distributed in 10 different bacterial species with a predominance of *S. aureus* with 32 (40.50%) followed by *Staphylococcus caprae* with 18 (22.78%), *Staphylococcus xylosus* with 8 (10.12%), *Staphylococcus simulans* with 7 (8.86%), *Staphylococcus epidermidis* with 5 (6.32%), *S. cohnii* with 4 (5.06%), *S. lentus* with 2 (2.53%), *S. hominis* with 1 (1.26%), *S. uberis* with 1 (1.26%), and *Streptococcus agalactiae* with 1 (1.26%). According to the genus, our investigation shows the importance of CNS as the most dominant bacteria at 45/47 (56.96%) followed by *S. aureus* at 32/79 (40.50%) and *Streptococcus* spp. at 2/47 (2.53%) (Table-4). In general, *S. aureus* was most often isolated from clinical mastitis cases, whereas CNS were most commonly found in SCM cases. SCM dominants (CNS) and streptococci were poorly isolated from clinical mastitis and no streptococcal species were isolated from SCM samples either. Overall, the survey determined that the frequency of isolated bacteria varies from one mastitis to another and from one region to another. We recorded 39.24% (31/79) frequency for clinical mastitis versus 60.76% (48/79) frequency for SCM. Depending on the region, we found 31.64% (25/79) frequency for Tebessa, followed by Souk Ahras at 24.05% (19/79), Guelma at 22.38% (18/79), and Skikda at 21.51% (17/79) (Table-4).

**Table 4:** Distribution and frequencies of isolated bacterial species.

Regions	Type of mastitis	*Stap. aureus*	Bacterial species isolated from mastitis									Number of germs isolated by mastitis	Number of isolated germs by region

			Coagulase-negative staphylococci							*Strept.*			
	
			*Stap. caprae*	*Stap. xylosus*	*Stap. stimulus*	*Stap. epidermidis*	*Stap. cohnii*	*Stap. lentus*	*Stap. hominis*	*Strep. uberis*	*Strep. agalactiae*		
Tébessa	Clinical mastitis	10	0	0	0	0	0	1	0	0	0	11	25/79 (31.64%)
	Subclinical mastitis	1	5	1	4	2	0	0	0	1	0	14	
Souk Ahras	Clinical mastitis	6	0	0	0	0	0	0	0	0	0	6	19/79 (24.05%)
	Subclinical mastitis	0	6	3	1	1	1	1	0	0	0	13	
Guelma	Clinical mastitis	7	0	0	0	0	0	0	0	0	0	7	18/79 (22.78%)
	Subclinical mastitis	1	4	2	1	1	1	0	1	0	0	11	
Skikda	Clinical mastitis	7	0	0	0	0	0	0	0	0	0	8	17/79 (21.51%)
	Subclinical mastitis	0	3	2	1	1	2	0	0	0	1	9	
Total	Clinical mastitis	30	0	0	0	0	0	1	0	0	0	31/79 (39.24%)	79
	Subclinical mastitis	2	18	8	7	5	4	1	1	1	1	48/79 (60.76%)	
	Mastitis (clinical, Subclinical) (%)	32/79 (40.50)	18/79 (22.78)	8/79 (10.12)	7/79 (8.86)	5/79 (6.32)	4/79 (5.06)	2/79 (2.53)	1/79 (1.26)	1/79 (1.26)	1/79 (1.26)	79	
			45/79 (56.96)							2/79 (2.53)			

*Stap.*=*Staphylococcus, Strept.*=*Streptococcus*

Finally, all samples from clinical mastitis and positive CMT gave positive results in bacteriological examinations.

## Discussion

Clinical examination of udders showed that dairy goats of various ages (primiparous and multiparous) were susceptible to mastitis. Applying CMT to healthy udder halves revealed the presence of SCM in primiparous and multiparous goats.

Bacteriological examinations revealed the characteristics of the etiology of clinical mastitis, where *S. aureus* was the most commonly isolated bacteria from the udder (40.50%) and streptococci (6.25%) were the least isolated. This result is consistent with the majority of studies that report that, unlike bovine mastitis, streptococci are very rarely the cause of mammary infections in goats [18-25].

SCM is difficult to detect with milk and udders that appear healthy. However, it can be suspected if there is a decrease in milk production [26]. SCM can then be revealed using a CMT test or Teepol test. This test has been used for more than 40 years in several countries [27] and remains the most used and successful test in dairy cows for detecting SCM [3,28,29]. It gives a qualitative answer on the status of each quarter of the udder (healthy or infected) and makes it possible to select which animals to sample from during study on mastitis [30,31]. It has the advantage of being inexpensive and providing an immediate response [32].

Regarding SCM in the current study, CNS were the most commonly isolated bacteria in seemingly normal goats (95.75%) and *S. aureus* was the second most common (4.25%). Similar results have been reported in other studies: Lerondelle and Poutrel found 76.24% for CNS versus 17.8% for *S. aureus*. El Idrissi recorded 42.9% for CNS and 13% for *S. aureus*, while Ferrer showed 53.2% for CNS and 13.5% for *S. aureus*, and Contreras identified 30.3% for CNS as well, while Bergonnier isolated 60-90% of CNS [21, 26,32-34].

In contrast to our results, *Enterobacteriaceae*, *Pseudomonas*, *Corynebacteria*, *Bacillus*, *Mycoplasma*, and *Erysipelothrix* have been isolated in several studies [19,20,25,32]. Regarding the lack of isolation of enterobacteria in our investigation that may have been related to the physiological and anatomical character of the goat: The droppings of goats are hard and dry which reduces the contamination of the udder, by fecal germs, in this species [33]. In addition, the teat canal is smaller, and therefore, contamination from the environment is more difficult.

Overall, staphylococci mammary infection in goats is due to the presence of these commensal germs of the mammary integument [34-39]. Therefore, contamination usually occurs during milking operations and not from the environment. Other studies have confirmed the significance of staphylococcal strain present in the etiology of SCM in sheep and cows [40-43].

Finally, the higher prevalence of intramammary infection with *S. aureus* can cause a public health problem in food safety.

This variation in the prevalence of mastitis (clinical and subclinical) could be attributed to the definition of infection, which is variable from one author to another [44], and to the use of different diagnostic methods (CMT and bacteriological examination SCCs). In sheep, the majority of authors propose a point threshold allowing the best “instant” discrimination between “healthy” and “infected” udders or half-udders. The study of Bergonier *et al*. (1994) provides a dynamic model describing whole lactations, where the best decision rule was as follows: A half-udder is “healthy” if all its SCCs except one are <500 * 10^3^ ¢/mL, “infected” if <3 SCC are >1000 * 10^3^ ¢/mL, and “doubtful” in all other cases. The overall value of this rule is 79.2% [45]. Bourabah *et al*. reported that the high percentage of the SCM could be due to a lack of hygiene and to the practice of traditional breeding of extensive type, which favors diseases [9].

## Conclusion

The present study, conducted on multiple dairy goat farms, has shown the prevalence of clinical mastitis with an average infection level of 40.50% versus 59.49% for SCM. It is not possible to avoid mastitis completely. However, we suggest that to reduce the incidence of mastitis in dairy goat farms in the study areas, it is essential to present the information to farmers on the causes, the economic and health consequences, as well as the control of mastitis should imply the clinical examination of the udder, and a systematic and early screening of these latent affections through a quick and reliable test, such as CMT. As well as, a bacteriological examination should be performed to identify the pathogens responsible for these infections to limit the transmission of infections and propose treatment or, if necessary, reform.

## Authors’ Contributions

ZG designed and carried out the research study along with AEG. ZD and MB helped with the design and correction of the manuscript. All authors read and approved the final manuscript.
